# Doctor competencies, patient expectations and healthcare context: connecting communication to satisfaction and trust

**DOI:** 10.1016/j.clinsp.2026.100903

**Published:** 2026-03-20

**Authors:** Carlos Frederico Confort Campos, Clarice Rosa Olivo, Gabrielle Leite Silveira, Milton de Arruda Martins, Patricia Zen Tempski

**Affiliations:** a Center for Development of Medical Education, Universidade de São Paulo, São Paulo, SP, Brazil; b The Centre for Medical and Health Sciences Education, University of Auckland, Auckland, New Zealand

**Keywords:** Health communication, Patient satisfaction, Trust, Physician-patient relations, Clinical competence, Patient expectations, Healthcare

## Abstract

**Objective:**

To investigate which factors mediate the association between doctors’ communication behaviours and patients’ satisfaction with care and trust placed in their doctors.

**Methods:**

Qualitative descriptive study conducted in a tertiary teaching hospital in São Paulo, Brazil, with 60 patients enrolled in the preoperative risk assessment clinic. They underwent a semi-structured interview, which was analysed using a reflexive thematic analysis approach.

**Results:**

Patients' satisfaction with care and trust placed in their doctors were analysed. The main themes identified as mediating factors were doctors' competencies, patients subjectiveness, and healthcare context.

**Conclusion:**

Better understanding the mediators involved in the association between communication and patients’ satisfaction and trust allows the creation of specific educational actions targeted at doctors.

**Innovation:**

We use a qualitative approach to identify mediating factors and shed light on how what happens in the “doctor’s office” can mediate the connection between communication, satisfaction and trust.

## Highlights

• Patients trust and satisfaction with healthcare are closely linked.
• Patient-doctor communication relates to patient satisfaction and trust with care.
• Factors mediate the relationship between communication, satisfaction, and trust.
• Doctors clinical and relational competencies are important mediators.
• Patients expectations and healthcare context play a critical role as mediators too.

## Introduction

In the healthcare context, the body of research showing a link between the good use of communication skills and positive health results is extensive.^1^, ^2^, ^3^ A critical skill for medical practitioners,^4^ effective communication between doctors and patients can lead to better patient outcomes, ranging from objective to more subjective measurements.^5^^,^^6^

**Fig. 1:**
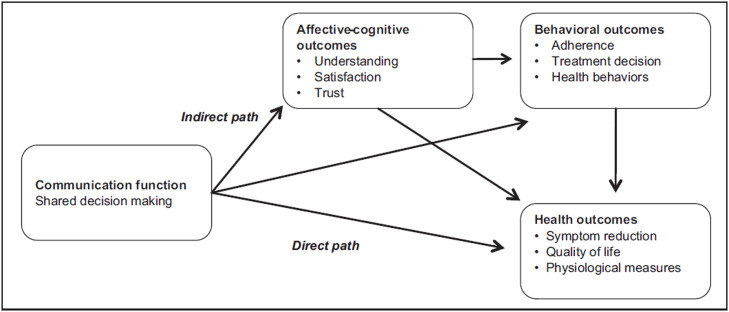
Conceptual model adapted by Shay and Latafa connecting communication with health outcomes. Reproduced from Shay and Latafa [awaiting permission from SAGE Publications].

Different models have been proposed to explain the connection between communication and good health outcomes. A model, created by Street et al.,^7^ and later adapted by Shay and Latafa,^8^ using Kreps et al.^9^ patient-centric health outcomes categories, proposes two different paths. A rare and direct one, in which communication affects directly more definitive health outcomes; and a more common, indirect one, in which communication affects health through intermediate outcomes, such as affective-cognitive (e.g., satisfaction and trust), and behavioural outcomes (e.g., adherence and adoption of health behaviours)^8^ (Fig. 1). This model has been used commonly for several reasons. First, it makes sense: a communicative interaction (or even a few) is unlikely to directly affect a health outcome. Second, studies have already shown the connection of these ‘middle’ outcomes with health outcomes (e.g., a patient with diabetes^10^ or chronic back pain.^11^) Finally, studies have also demonstrated the link between doctor-patient communication and affective-cognitive outcomes.^12^^,^^13^

Although the model paints a good picture to explain the communication-health outcomes connection, it describes the ‘bigger picture’. What happens in the doctor-patient interaction – more importantly, what is communicated within that space – seems to have an important influence on the final health outcomes.^14^^,^^15^ What happens in the doctor’s office produces the proximal link between communication and affective-cognitive outcomes, shown in Fig. 1.

Trust and satisfaction have been the subject of extensive research. Their significance derives from the fact that they work as proxy measures of the doctor-patient relationship. Although both constructs are distinct and independent,^16^ their connection is recognised in the literature.^17^^,^^18^ That connection is also intuitive: in a health interaction, a patient who is satisfied with the care they are receiving will probably trust their doctor. Conversely, a patient who does not trust their doctor would hardly be satisfied with the care they are providing.

Trust and satisfaction need to be in the foundation of a healthcare relationship to make it productive for both parties.^19^^,^^17^ A satisfactory interaction between doctors and patients can help strengthen the therapeutic relationship, leading to trust, and consequently to more accurate diagnoses and compliance.^20^ And communication plays an important role in achieving those results.

Better understanding what is involved in the connection between communication and patient satisfaction and trust placed in their doctor will further inform us on the specifics of the indirect path proposed by Shay and Latafa.^8^ This will lead to greater recognition of the importance of communication in the healthcare setting, allow the design of more targeted training interventions focusing on healthcare students and clinicians, and, ultimately, improve patients’ health and healthcare experience using good communication.

Usually, quantitative methods are preferred as models for investigating mediation analysis. Statistical mediation models verify the connection between an independent variable to a dependent one, through an intervening variable.^21^ However, because complex processes are difficult to dissect, valuable information can come from different methods.^22^ Qualitative methods can offer further insight into the mediation processes through patients’ perspectives.^23^^,^^24^ Through reflexive thematic analysis of interview data, we constructed themes that help explain the mediation processes through participants' experiences.

Thus, this study aims to put a ‘magnifying lens’ on the framework in Fig. 1, by asking the following research question: which factors mediate the association between doctors’ communication behaviours and patients' satisfaction with care and trust placed in their doctors?

## Methods

### Ethics

Our study was approved by the Ethics Committee of the School of Medicine of the University of São Paulo (*Comissão de Ética para Análise de Projetos de Pesquisa* ‒ CAPPesq) and by the National Committee of Ethics in Research of the Ministry of Health of Brazil (*Comissão Nacional de Ética em Pesquisa* ‒ CONEP), protocol number 2.825.441. All participants read and signed the informed consent form.

### Study design

We undertook a qualitative descriptive study to explore participants' perceptions of their doctor’s communication. This article is a report on the qualitative arm of a mixed-methods study that focuses on the relationship between communication skills and doctor-patient productive interactions, previously published.^25^ This study followed the Consolidated Criteria for Reporting Qualitative Research (COREQ)^26^ to promote transparency and completeness in the reporting of qualitative methods and findings.

### Context

The study was conducted in a preoperative risk assessment clinic setting. The clinic is part of *Hospital das Clínicas*, a tertiary teaching hospital associated with the University of São Paulo in São Paulo, Brazil. It was chosen for the study because it is the main clinic for preoperative risk assessment in the hospital.

### Sampling approach and sample population

Data was collected by one of the authors (CC), who does not have any connection to the studied clinic nor is a doctor at the studied hospital. It occurred between April and June 2019. Patients with medical appointments on the study days were approached to rule out any exclusion factors. All eligible patients were invited to the study.

Inclusion criteria were patients enrolled in the preoperative risk assessment clinic with planned procedures. Exclusion criteria encompassed any significant cognitive, visual, or auditory impairment.

Participation in the study was voluntary. It was made clear to participants that participation or not in the study would bear no relation to their clearance for their procedure nor any health treatment. No compensation was offered for participating.

### Data collection

After informed consent, the patient underwent their previously booked consultations with their doctors, where they would be assessed for clearance for the procedure. Regardless of outcomes and immediately following the consultation, patients were asked to take part in an interview regarding their doctor’s communication, their satisfaction with the care received, and the trust they placed in their doctor. The interview was conducted by one of the authors (CC) in a private consultation room. The interview was recorded in audio. We also collected patients' age and gender as sociodemographic data.

### Interview script

The interview followed a semi-structured script comprised of the following questions:

1. Some medical consultations make us feel good, others not that much. How did you feel by the end of this consultation? Why?
2. What made you trust this doctor?
3. What made you not trust this doctor?
4. Can you think of something that would have made you feel more satisfied with the consultation, had it happened? Can you describe it?

Those four questions guided the interviews, with subsequent questions being asked based on participants' responses. The script was developed by the authors based on the aims of the study.

### Data analysis

A research team member (CC) listened to and transcribed the audio recordings of the interviews. The transcripts were checked to ensure accuracy.

Interview transcripts were analysed using reflexive thematic analysis. Through this method, one can identify, analyse, and report patterns ‒ or themes – within data. Specifically, we used the reflexive thematic analysis approach.^27^ We identified themes inductively, driven by the research question, at a semantic level, within a realist/essentialist paradigm.

Following Braun and Clarke’s reflexive thematic analysis, the analytic process involved six recursive phases: familiarization with the data; generating codes; constructing initial themes; reviewing and developing themes; defining and naming themes; and producing the analytic narrative using illustrative data extracts. As emphasized by the authors, this process is not linear but reflexive and iterative, allowing the researcher to move back and forth across phases as analytic insights develop. Within this approach, themes were conceptualized as patterns of shared meaning across the dataset that are underpinned by a central organizing concept and are relevant to addressing the research questions, irrespective of prevalence or the number of participants contributing to them.

To ensure reflexivity and mitigate researcher bias, data were analysed independently by three authors: CC (a male general practitioner, and PhD candidate in medical education), CO (a female physiotherapist, and researcher in medical education) and GLS (a female psychologist and PhD candidate in medical education). All three authors had experience in clinical, teaching, and research settings. Finally, the analyses were discussed, including a fourth member of the team – PT, a female paediatrician with habilitation and research experience in medical education – to achieve consensus among the research team. Illustrative quotes are reported verbatim. The study was conducted in Brazilian Portuguese. The quotes were translated into English by the authors for the publication.

The research team took the methodological decision of analysing both satisfaction with care and trust placed in the doctor combined. Although we acknowledge that those are two different concepts, our decision was based on their close connection. Usually, it is very difficult for a patient to trust a doctor if they are not satisfied with the care they are receiving. The opposite is even more rare, for a patient to be satisfied with the care provided by a doctor they don’t trust.

### Findings

Our study included 60 participants, with 36 (60 %) females and a mean age of 61-years old. Patients were being assessed for a variety of surgical procedures (i.e., Ophtalmology, Urology, General Surgery). Forty-nine (81.7 %) were cleared for the procedure. We did not detect any pattern between clearance for procedure and patient perspectives.

We identified three overarching themes that function as mediators of the association between doctors’ communication behaviours and the combination of patients’ satisfaction with care and trust placed in their doctors. The themes were: doctors’ competencies, patients subjectiveness, and healthcare context. Under each of those themes, we identified a set of subthemes, as described below.

### Doctors’ competencies

Doctors’ communication is a critical competency. However, this theme relates to other important competencies that doctors develop and demonstrate during their interaction with their patients. Our subthemes derive from two of those competencies: relational and clinical.

#### Relational competency

The satisfaction-trust combination derives significantly from the relationship established between patient and doctor. The more developed the doctor’s relational competency, the easier it is for patients to build the combination.

Under that subtheme, patients mentioned the opportunity to express themselves during the medical encounters – either positively, negatively (when they felt they couldn’t), or as something novel:

- ‘She listened to me. I think that is very important. She didn’t act as she knew it all already.’ – female, 54-years-old.
- ‘I would never trust him. Because he doesn’t know me, he has never seen me before, and he didn’t even want to talk, or anything.’ – female, 81-years-old.
- ‘I trusted everything. Because I trusted him. There are some doctors who barely look at your face. He looked me in the eye. He talked to me as if we were equals.’ – male, 54-years-old.

The relational competency was identified when patients felt their doctors were being clear or even sincere in their explanations:

- ‘The way he talks to us, being kind. I am sure he is trustworthy, explaining everything clearly.’ – male, 65-years-old.
- ‘Oh, the candour, right? She was very clear to me.’ – female, 50-years-old.

Patients also commented on their doctor’s relational competency when they appear to be interested in their patients, by asking questions, answering them, or mentioning negative previous experiences:

- ‘I thought he is a good clinician, he conducted a very nice consultation, asked all the questions, everything was right. And it is so good when someone cares about us, right?’ – female, 69-years-old.
- ‘Because she answers when I ask a question.’ – female, 46-years-old.
- ‘She asked everything she wanted to know, because there are some doctors that barely ask you any questions.’ – female, 51-years-old.

#### Clinical competency

Patients want their doctors to be competent. Although we know there are many different (and important) competencies a doctor must have, patients usually mean they want their doctors to be clinically competent. In that sense, we understand clinical competency as a characteristic of a healthcare professional that relates specifically to their clinical practice. We identified that the clinical competency of doctors is also associated with how the satisfaction-trust combination is created for our participants:

- ‘The way he asks questions and does the medical assessment, within the medical knowledge that he has.’ – female, 61-years-old.

The clinical competency is illustrated by specific clinical acts, like taking a history or performing a physical examination:

- ‘Oh, because not only did he ask all the questions for the surgery, but he also asked about my previous medical consultations. He really cared about me.’ – female, 70-years-old.
- ‘He checked me thoroughly. He examined me. With both questions and the medical equipment.’ – male, 53-years-old.

Finally, as was the case for satisfaction with care and trust placed in the doctor, we also observed that patients mentioned relational and clinical competencies as a combination, without clear boundaries between the two of them:

- ‘Because I noticed that the doctor asked me a lot of questions, she cares about my health, she cares about what we need to do now, that is the surgery.’ – female, 26-years-old.

### Patients’ subjectiveness

As our second theme, we identified that an important part of what is communicated in the patient-doctor interaction relates to the patient’s subjectiveness, which is critical to the existence of the satisfaction-trust combination. Patients create meaning through a mix of what has been said by the doctor, what is understood, and their previous experiences (in daily life and in the healthcare relationship context). Thus, we named our subthemes ‘perceptions’ and ‘expectations’.

#### Perceptions

Firstly, we describe the perceptions participants had regarding behaviours that are to be expected (although not always found) in the interaction between two ordinary people, such as respect, politeness, and kindness:

- ‘Because the doctor conducted a very good consultation, she was very polite and kind.’ – female, 77-years-old.
- ‘Kind, considerate. I was consulted by someone very kind.’ – male, 64-years-old.
- ‘I didn’t like him at all. He cut the conversation short and was impolite. Rude, very rude, you know?’ – female 81-years-old.

In addition to these subjective but easily recognisable behaviours, participants also mentioned some features of their doctors that are more difficult for them to assess. However, participants mentioned them as ‘clearly recognisable’ characteristics of their doctors, such as clinical competence, full understanding of their needs, or even joy and love:

- ‘Not only is she an excellent doctor, but the treatment she prescribed is also good.’ – male, 58-years-old.
- ‘Oh, by the questions he asked. He understood everything that I told him.’ – female, 70-years-old.
- ‘The joy and love he has inside in his heart.’ – female, 68-years-old.

Those subjective perceptions led to patients’ feelings of ease and being taken care of.

- ‘I felt taken care of.’ – female, 63-years-old.
- ‘He made me feel at ease.’ – female, 49-years-old.

#### Expectations

The expectations patients built around their health had a very close relationship with the satisfaction-trust combination:

- ‘Because the doctor was considerate, kind, and said exactly what I wanted to hear from him.’ – female, 82 years old.

As our setting was a preoperative risk assessment clinic, patients’ main expectations were to be cleared for their surgical procedures. They expressed those relationships in both positive and negative situations:

- ‘Because it fulfilled my expectation to be cleared for surgery.’ – female, 45-years-old.

Participants mentioned other expectations, not only related to clearance for surgery:

- ‘Because my expectation of a full explanation of the procedure I will do was met.’ – female, 75-years-old.
- ‘He consulted me and met my expectations.’ – female, 63-years-old.

Expectations were also noted when participants were asked to state what they would like to have happened differently:

- ‘If I was told I wouldn’t need general anaesthesia anymore.’ – female, 77-years-old.
- ‘If the doctor told me: ‘you can go, you are cleared, and you can go back to [participant’s hometown]’.’’ – female, 74-years-old.
- ‘If he told me I don’t have a hole in my heart anymore.’ – female, 61-years-old.

Finally, we identified that patients’ expectations were met when they mentioned that their doctors were ‘on their side’, wishing them the best. These positive statements also contributed to the satisfaction-trust combination:

- ‘Because I felt something positive from him, that everything would be fine.’ – female, 49-years-old.
- ‘He wished me good luck on the surgery.’ – male, 60-years-old.

### Healthcare context

The context in which a relationship is developed is critical for its participants to create meaning of what is communicated. The communicative interaction between patients and doctors is no different: it is also influenced by the healthcare context. And so is the patient perception of the satisfaction-trust combination. Under the healthcare context, we identified the following subthemes: reference hospital, teaching hospital, faith in God, medical profession, and work under supervision. We understand that the five subthemes are different forms of ‘institutions’ which, in their own way, help patients create meaning of what was communicated in the interaction.

#### Reference hospital

We conducted the study in *Hospital das Clínicas*. It is a reference hospital – or a referral hospital – for patients who need care that can’t be delivered in primary or secondary care health units. In addition, it is a reference, a landmark to the general population. It is nationally recognised as a healthcare service where ‘all your demands will be sorted’. The word in Brazilian Portuguese for ‘reference’, ‘landmark’ and ‘referral’ is the same: *referência*. Some participant quotes illustrate this mix of ‘reference’ characteristics:

- ‘We are in a renowned hospital. It is an almost worldwide landmark.’ – male, 47-year- old.
- ‘Just by the fact that he is a doctor who works in this hospital, I trust him completely.’ – male, 58-years-old.

#### Teaching hospital

Our study setting is not only a reference hospital but also a teaching hospital. It provides training for doctors, nurses, and several other health professionals in the undergraduate, postgraduate, or continuous professional education contexts. That characteristic was also part of our participants' remarks:

- ‘I usually trust a little bit less in a teaching hospital, but lately, this hospital looks a bit different.’ – female, 46-years-old.
- ‘He went to talk to his ‘boss’ because he is a trainee.’ – female, 75-years-old.

#### Faith in god

Participants commented on their faith in God as an important factor associated with their satisfaction-trust combination. In Brazilian culture, God is frequently mentioned as a protective factor in healthcare. Of note, in the Brazilian context, when God is cited, it usually means the Christian God since Christianity is the main religion professed in Brazil. The following quotes exemplify this feature:

- ‘Firstly, I trust God, then her [the doctor].’ – female, 68-years-old.
- ‘I know that they care for me. He [the doctor] is kind to me. And I trust in God.’ – female, 74-years-old.

#### Medical profession

The clinical practice of doctors is also linked to the satisfaction-trust combination by our participants. However, in our study, it became clear that it was not just about the practice of a single doctor, as much as the ‘medical institution’ as a craft:

- ‘Because doctors are quality trained professionals and work with such love.’ – male, 54-years-old.
- ‘We always trust in doctors' consultations, right?’- female, 46-years-old.

This institutional aspect appears to be robust, leading to a feeling of unquestionable professional capability of doctors, as per the quote:

- ‘I frequently go to the doctors, and that is why they are there, right? So, I’ll neither trust nor distrust them. When we don’t trust a doctor, we don’t even see them. So, I know he is a good doctor.’ ‒ female, 46-years-old.
- ‘We must trust the [medical] professional because if he is there, he is competent to do the job.’ ‒ female, 50-years- old.

#### Work under supervision

The doctors’ work is important to building the satisfaction-trust combination. However, the fact that doctors are not alone in handling their patients’ care also contributes to the creation of satisfaction and trust.

- ‘I know that there is always the endorsement of other doctors, that is why I feel safe with any doctor here.’ – female, 45-years-old.
- ‘The fact that he leaves to discuss with his ‘boss’ outside.’ – female, 48-years-old.

## Discussion and conclusion

### Discussion

We found that doctors’ competencies, patients' subjectiveness, and the healthcare context function as mediating factors in the association between doctors’ communication and patients’ satisfaction with care and trust placed in their doctors. As subthemes of doctors’ competencies, we found relational and clinical competencies; under patients' subjectiveness, we identified perceptions and expectations; and as subthemes of the healthcare context, we proposed reference hospital, teaching hospital, faith in God, medical profession, and work under supervision.

On the doctors’ end, we identified two mediators, the clinical and the relational competencies. These mediating factors to patients’ trust and satisfaction are expected, since patients are ‘hopelessly’ dependent on their doctors’ competencies,^28^ to be diagnosed or treated by them. Our findings corroborate the idea that those two competencies are strongly linked to trust and satisfaction, since both help in the establishment and intensification of the patient-doctor relationship.^3^^,^^29^^,^^30^

An important distinction must be made regarding relational competency, as it risks becoming tautological when thought solely as a component of communication. Although relational competency is deeply embedded in communication ‒ and indeed largely constituted by it ‒ we aim to focus on the doctor’s ability to establish and sustain a therapeutic relationship. Within a highly specific context such as the preoperative clinic, we found this competency as a mediator of the combination of satisfaction and trust, rather than merely reiterating communication skills under a different label. Thomas et al. (2020) also found that mediation with general practitioners.^31^

Due to our decision to analyse both satisfaction and trust as a monolithic aspect, we also considered it important to hypothesize how each theme would be related to each construct. Our perspective is that clinical competency – more objectively perceived – tends to affect more trust, while relational competency must influence more satisfaction, owing to its more subjective aspect.

However, this division between the clinical and relational competencies is artificial and cannot be observed in clinical practice. When communicating with patients, clinicians use both competencies simultaneously.^32^ A study reports that the mental load of medical students is not increased when they simultaneously carry out a more ‘clinical’ task (e.g., venous puncture) and a ‘communication’ task (e.g., taking history) when compared to when they just perform venous puncture.^33^ It shows that they make use of both competencies ‘as one’. Further, in the modern healthcare setting, patients are seen as more active subjects in their own healthcare. Being ‘partners’ in their own care,^34^ reinforces how connected these two competencies are.

The patient’s subjectiveness was also found to be an important mediator between doctors’ communication and patients trust and satisfaction. Included in that there, we identified the patients’ expectation performing a mediating factor. The connection has been shown by Hall et al.’s^35^ description of trust, where the one who trusts expects positive outcomes coming from the trust placed. As for satisfaction, Goldzweig et al.^36^ state that satisfaction comes from the difference between what the patient expected to get and what they actually got in terms of healthcare delivery. Studies on more acute conditions (i.e., cancer or orthopaedic surgery) show the importance of setting appropriate and realistic expectations to achieve better patient satisfaction.^37^^,^^38^ In our study, even unmet (or unrealistic) expectations, such as not needing surgery anymore, lead to patients’ satisfaction and trust.

We identified patients’ perceptions as an important mediating factor. A study by Ng and Luk shows that patients report more satisfaction with friendlier, more affective, and caring professionals.^39^ This can vary in different countries and cultures. In Brazil, both quantitative^40^ and qualitative^41^ research have shown that patients tend to expect their healthcare providers to be more effective. Patients tend to frequently comment on their doctor’s capabilities,^39^ even if they are not in the best (technical) position to assess them.^28^ Something similar also applies to the more affective perceptions patients have of their doctors. One cannot be absolutely sure that they are dealing with a caring professional. However, according to Betancourt,^42^ it does not matter if the perception is ‘true’ or ‘false’ – or if that is even possible to judge. Betancourt suggests that ‘perception is the reality’. Therefore, considering the healthcare context, what patients think of their doctors is their reality – regardless of any sort of ‘proof’.

Our final broader theme that works as a mediating factor between doctors’ communication and patients' trust and satisfaction is the healthcare context. Our study was conducted in Brazil, and some features are important to highlight. As discussed previously, the concepts of referral and reference hospital are blurred by users of the healthcare system in Brazil. Studies in Iran and China show that patients tend to prefer referral hospitals when choosing where to seek care.^43^^,^^44^ That is probably very similar in Brazil, in the local public health system, patients cannot make such a choice. Additionally, a Chinese study reported good satisfaction with referral hospitals.^45^

Another important mediating factor mentioned was the medical profession. Our understanding is that, for that aspect, the satisfaction-trust combination is more skewed towards the trust aspect. A survey from 2019 shows that doctors are the second most trustworthy profession worldwide.^46^ The same study reports that in Brazil, doctors come in third place, just behind teachers and scientists. It is an impressive figure considering the profession lost some of its prestige over the last five decades.^47^ Those two factors feed into each other. The doctors’ ‘personal trust’ helps build the hospital’s ‘institutional trust’. Conversely, the hospital’s ‘institutional trust’ instils some ‘personal trust’ into its doctors.^48^

Finally, we consider it important to discuss the identification of faith in God as a mediating factor. In Brazil, around 81 % of the population professes a Christian religion.^49^ A study by Lucchetti, Almeida, and Granero reported that for patients with chronic kidney disease, satisfaction with care was positively associated with patients’ levels of religiosity/spirituality.^50^ On the trust end of the combination, Ventres and Dharamsi argue that the concept of faith is basically trusting something or someone.^51^ They state that faith has been and will always be a part of medicine. We advocate that this trust, placed in God, is transferred to the doctors in a somewhat similar fashion as the process that occurs between the hospital and doctors.

### Limitations and strengths

Our study presents some limitations. First, we analysed the mediating factors as a combination of satisfaction and trust. As we stated previously, we recognise their difference. However, we intentionally decided to combine them into the same analysis, considering how strongly connected they are. Further, the fact that we chose a preoperative risk assessment clinic as our study setting may have facilitated the combination of trust and satisfaction, especially when patients were cleared for surgery. A potential bias might be expected as patients would hope for doctor approval and procedural clearance. That probably explains the skewed, overwhelming majority of positive responses we had from participants, which shows an expected social acceptance bias.

Additionally, having executed the study in such a specific, high-stakes context can limit the generalisability of our findings. Finally, our participants underwent consultations with a significantly qualified cohort of doctors (second-year internal medicine residents), which might have brought an effect of similarity and high performance. We could see different findings in a more heterogeneous sample of doctors.

We have also identified some strengths of the study. We looked into doctors’ communication, but analysed the patient’s perspective of satisfaction and trust. We consider that it is important (and somewhat recent) as it sheds light on the ‘flip side’ of patient-centred communication in high-stakes, single-encounter, gatekeeping settings. Finally, some of our identified mediating factors can be improved by acting on the main factor that can be changed in the ‘equation’, which is the doctor. The doctors’ competencies and behaviours can strongly influence patients’ subjectiveness and, ultimately, the healthcare context.

## Conclusion

In the association between doctors’ communication and the combination of patients’ satisfaction with care and trust placed in their doctors, we identified doctors’ competencies, patients subjectiveness and the healthcare context as mediating factors, in high-stakes, single-encounter, gatekeeping settings. Satisfaction and trust are affective-cognitive outcomes that commonly connect doctors’ communication to more definitive health outcomes. Better understanding the mediators involved in the association facilitates the creation of educational actions targeted at doctors, considering specific cultural and contextual settings. These actions should aim to enhance their competencies, in turn, improving patients’ subjectiveness and, ultimately, the healthcare context.

## Data availability

The datasets used and/or analysed during the current study are available from the corresponding author upon reasonable request.

## Authors' contributions

**Carlos Frederico Confort Campos:** Conceptualization; data curation; formal analysis; investigation; methodology; project administration; resources; validation; visualization; writing-original draft; writing-review & editing.

**Clarice Rosa Olivo:** Formal analysis; investigation; validation; writing-review & editing.

**Gabrielle Leite Silveira:** Formal analysis; Investigation; validation; writing-review & editing.

**Milton de Arruda Martins:** Conceptualization; methodology; resources; supervision; writing-review & editing.

**Patricia Zen Tempski:** Conceptualization; methodology; project administration; resources; supervision; validation; writing-review & editing.

## Funding

This research did not receive any specific grant from funding agencies in the public, commercial, or not-for-profit sectors.

## Conflicts of interest

The authors declare no conflicts of interest.

## Appendix Supplementary materials

Image, application 1
